# Unique Double-Barreled Enzyme Makes Methionine the Hard Way

**DOI:** 10.1371/journal.pbio.0030054

**Published:** 2004-12-28

If a cell is a complex symphony of chemical reactions, its enzymes are the instruments through which this elemental music is played. Each reaction is catalyzed by a specific enzyme, whose uniquely shaped active site not only binds reactants, but, by forming weak and temporary bonds, coaxes them into new orientations with new partners, thus creating the products. Determining exactly how any individual enzyme accomplishes this task—which amino acids make up the active site, which bonds form where when enzyme meets substrate, which electrons switch partners as new bonds form—is the work of the structural biochemist. In this issue, Martha Ludwig and Robert Pejchal elucidate the structure of cobalamin-independent methionine synthase (MetE) from the bacterium Thermotoga maritima, and describe how it catalyzes the formation of the amino acid methionine.

Methionine synthases actually come in two forms, which use somewhat different mechanisms to accomplish the same task: transfer of a methyl group (CH3) from methyltetrahydrofolate to the terminal sulfur of homocysteine. The cobalamin-dependent form, MetH, relies on the cofactor cobalamin (vitamin B12), which pulls the methyl away at one active site, and then donates it at a second active site. Here, a central zinc atom binds and activates homocysteine, enabling it to attack the incoming methyl group that is attached to cobalamin. MetE, on the other hand, has no cofactor and only one active site, which sits at the junction of two eight-stranded barrels. The structure and sequence of these barrels indicate they arose through duplication of a primordial zinc-bearing, homocysteine-binding protein. This unique duplex now bears only one zinc atom, deep within the cleft separating the two barrels.

As in MetH, the role of the zinc is to bind homocysteine, but in MetE, this event also induces a conformation change around the zinc. The zinc and its coordinating partners form an umbrella; entering from the handle end, the homocysteine sulfur pulls the zinc toward it and turns the umbrella inside out. Methyltetrahydrofolate initially binds along the edge of the cleft, with the methyl group on the folate oriented far from the sulfur on the homocysteine, as can be seen in the research article's Video S1 (DOI: 10.1371/journal.pbio.0030031.sv001). There must be subsequent conformational changes within the active site that serve to bring the two substrates together and promote transfer of the methyl group. Exactly how methyltetrahydrofolate reorients within the cleft to complete the reaction is not yet clear. The reaction catalyzed by MetE proceeds slowly, at only 1%–2% of the speed of that catalyzed by MetH. One reason for this rather sluggish activity is that homocysteine, even when activated by binding to zinc, is much poorer than cobalamin at displacing the methyl group of methyltetrahydrofolate.

While MetE's unique active-site structure was made possible by gene duplication, the two barrels are no longer identical. Through evolution, the second, N-terminal, barrel has lost the ability to bind zinc or homocysteine, and indeed appears to contribute little to the active function of the enzyme. Nonetheless, this barrel may be necessary to temporarily isolate the substrates from solvent and to form the hydrophobic environment in which the reaction is more favorable. Further research may indicate more about the function of this unequal partner, and provide more detail on the exact atomic movements within the cleft at the moment of reaction.

